# Supplementary material for: Novel Cyclic Peptides from Lethal Amanita Mushrooms through a Genome-Guided Approach
Source: J Fungi (Basel). 2021 Mar 11;7(3):204. doi: 10.3390/jof7030204 (PMC7998459; doi:10.3390/jof7030204)
Supplement: Supplementary file 1 [file jof-07-00204-s001.zip › Additional file 1.pdf]

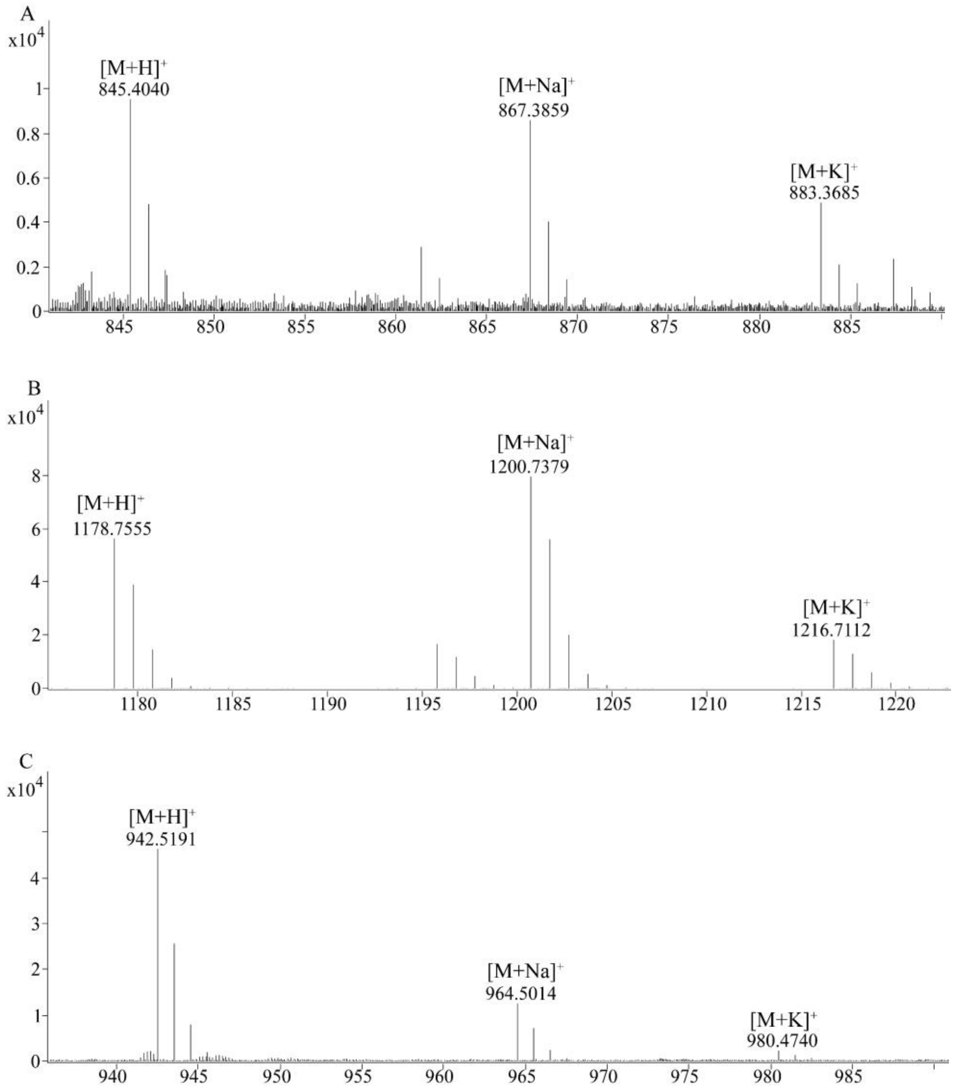

Fig S1. The monoisotopic masses of candidate new cyclic peptides in *Amanita rimosa.* The three molecules corresponded to the cyclized core peptide sequences of ISDPTAYP (A), FIPLGIITILP (B) and FPTRPVFP (C). The adduct ions of $[M+Na]^+$ and $[M+K]^+$ were shown in each section.

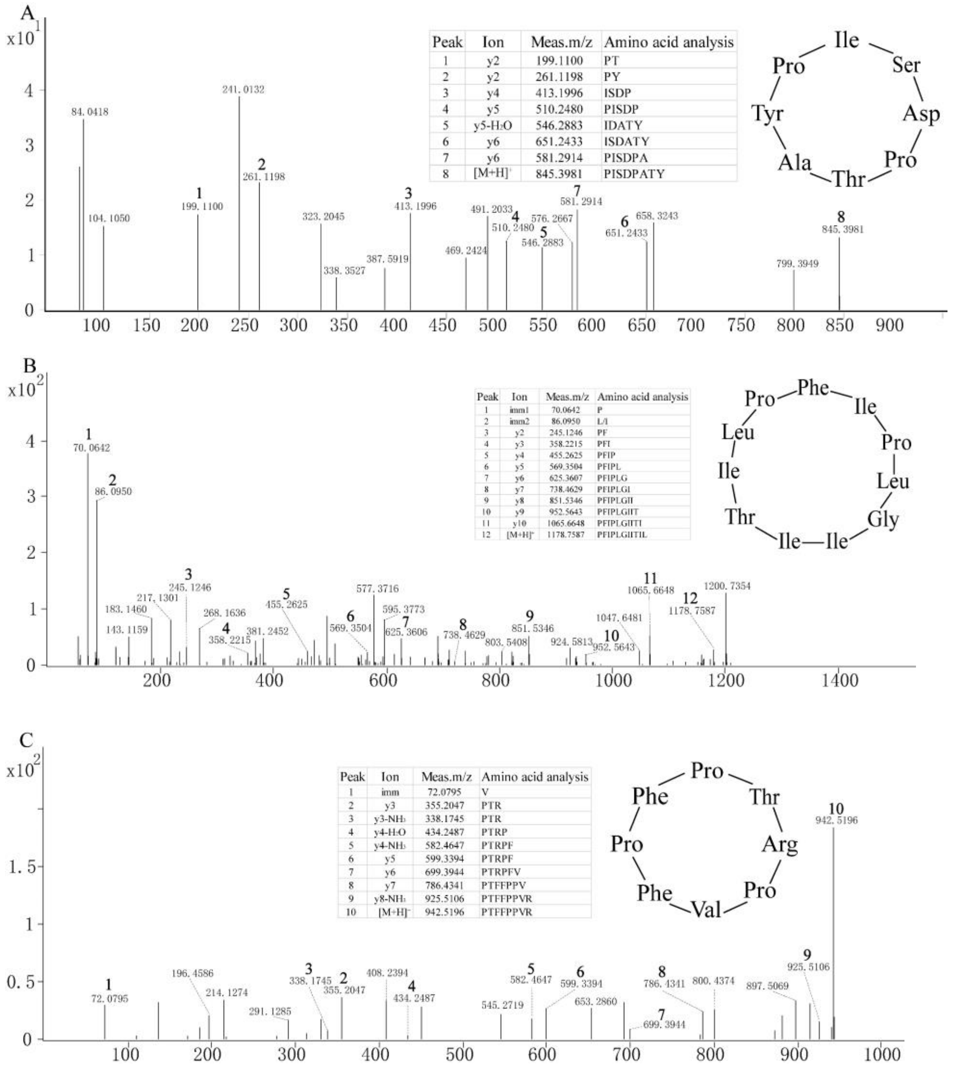

Fig S2. Tandem mass spectrometry of novel cyclic peptides in *Amanita rimosa*. Manual analysis of amino acid compositions of Cyclo(ISDPTAYP) (A), Cyclo(FIPLGIITILP) (B) and Cyclo(FPTRPVFP) (C), with inserted tables of matched amino acids; immonium ions were shown when present. The determined cyclic peptides were shown as circles.
