# Supplementary material for: Novel Cyclic Peptides from Lethal Amanita Mushrooms through a Genome-Guided Approach
Source: J Fungi (Basel). 2021 Mar 11;7(3):204. doi: 10.3390/jof7030204 (PMC7998459; doi:10.3390/jof7030204)
Supplement: Supplementary file 1 [file jof-07-00204-s001.zip › Additional file 2.pdf]

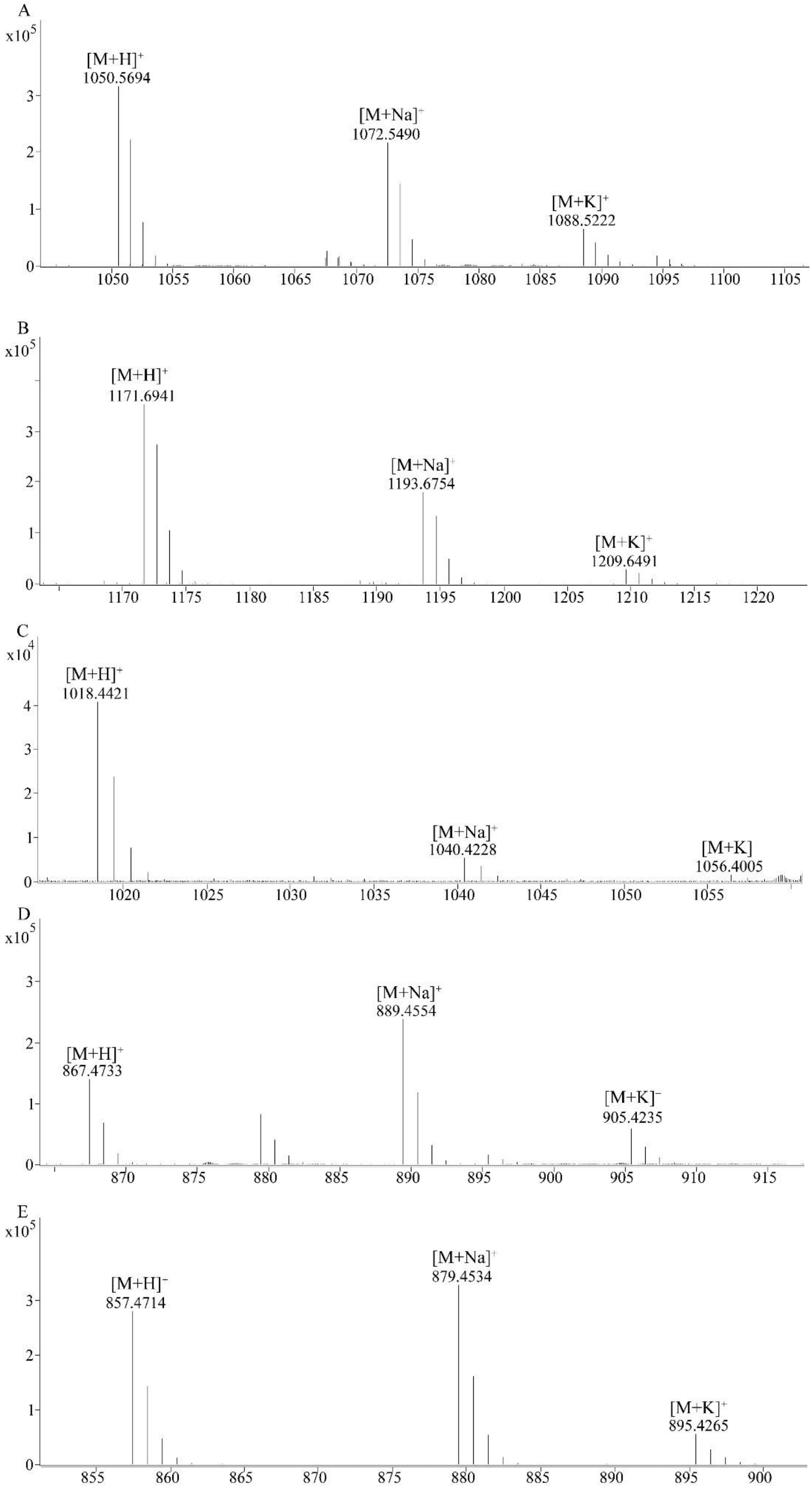

A
x10^5
[M+H]+
1050.5694
[M+Na]+
1072.5490
[M+K]+
1088.5222
B
x10^5
[M+H]+
1171.6941
[M+Na]+
1193.6754
[M+K]+
1209.6491
C
x10^4
[M+H]+
1018.4421
[M+Na]+
1040.4228
[M+K]
1056.4005
D
x10^5
[M+Na]+
889.4554
[M+H]+
867.4733
[M+K]−
905.4235
E
x10^5
[M+Na]+
879.4534
[M+H]−
857.4714
[M+K]+
895.4265

Fig S3. The monoisotopic masses of candidate new cyclic peptides in *Amanita pallidorosea.* The five molecules corresponded to the cyclized core peptide sequences of EFIVFGIFP (A), FVIIPPFIFP (B), YFFNDHPP (C), TIHLFSAP (D), and MHILAPPP (E). The adduct ions of $[M+Na]^+$ and $[M+K]^+$ were shown in each section.

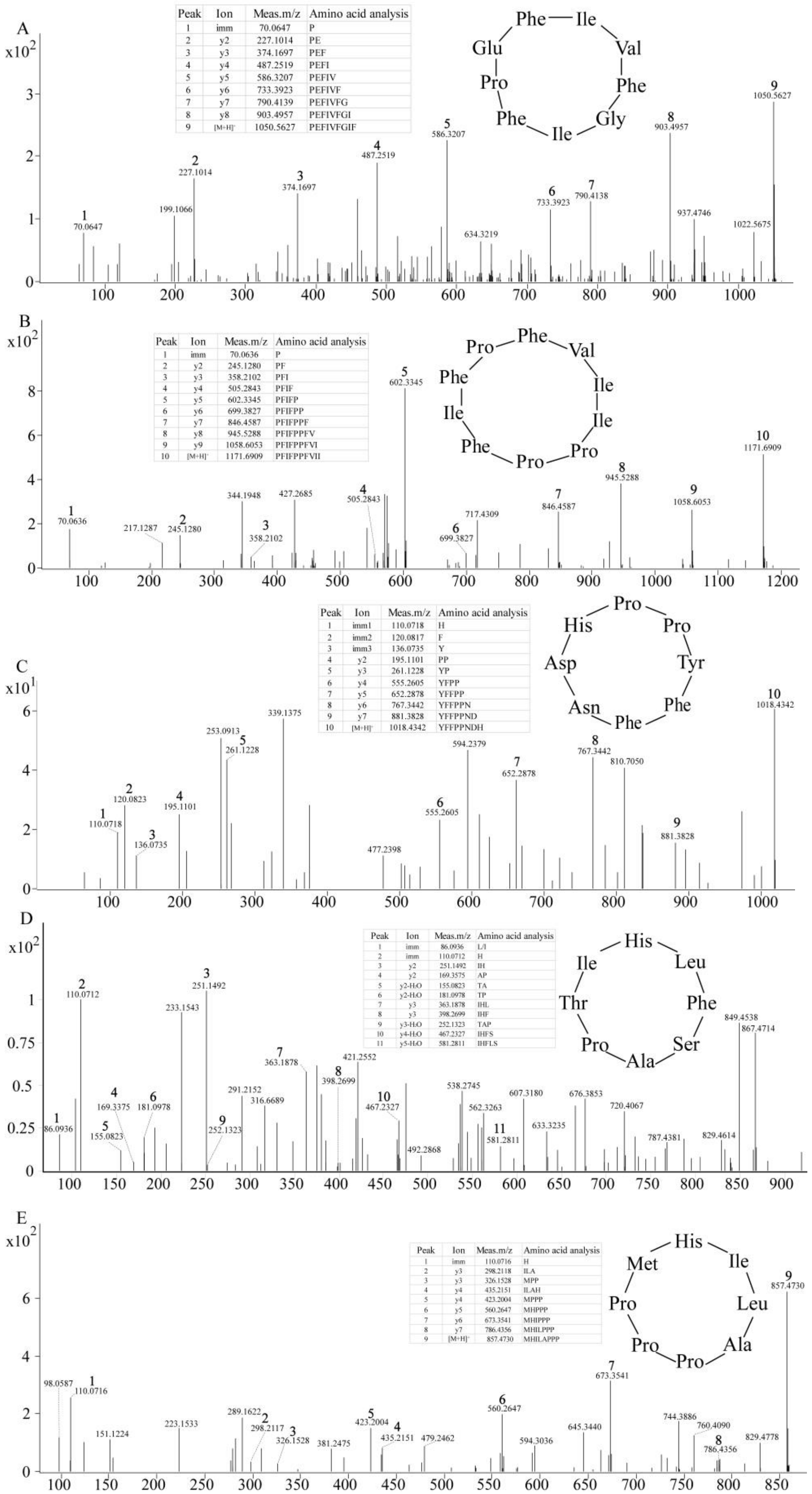

Peak	Ion	Meas.m/z	Amino acid analysis
1	imm	70.0647	P
2	y2	227.1014	PE
3	y3	374.1697	PEF
4	y4	487.2519	PEFI
5	y5	586.3207	PEFIV
6	y6	733.3923	PEFIVF
7	y7	790.4139	PEFIVFG
8	y8	903.4957	PEFIVFGI
9	$[M+H]^+$	1050.5627	PEFIVFGIF

Peak	Ion	Meas.m/z	Amino acid analysis
1	imm	70.0636	P
2	y2	245.1280	PF
3	y3	358.2102	PFI
4	y4	505.2843	PFIF
5	y5	602.3345	PFIFP
6	y6	699.3827	PFIFPP
7	y7	846.4587	PFIFPPF
8	y8	945.5288	PFIFPPFV
9	y9	1058.6053	PFIFPPFVI
10	$[M+H]^+$	1171.6909	PFIFPPFVII

Peak	Ion	Meas.m/z	Amino acid analysis
1	imm1	110.0718	H
2	imm2	120.0817	F
3	imm3	136.0735	Y
4	y2	195.1101	PP
5	y3	261.1228	YP
6	y4	555.2605	YFPP
7	y5	652.2878	YFFPP
8	y6	767.3442	YFFPPN
9	y7	881.3828	YFFPPND
10	$[M+H]^+$	1018.4342	YFFPPNDH

Peak	Ion	Meas.m/z	Amino acid analysis
1	imm	86.0936	L/I
2	imm	110.0712	H
3	y2	251.1492	IH
4	y2	169.3575	AP
5	y2-H_2O	155.0823	TA
6	y2-H_2O	181.0978	TP
7	y3	363.1878	IHL
8	y3	398.2699	IHF
9	y3-H_2O	252.1323	TAP
10	y4-H_2O	467.2327	IHFS
11	y5-H_2O	581.2811	IHFLS

Peak	Ion	Meas.m/z	Amino acid analysis
1	imm	110.0716	H
2	y3	298.2118	ILA
3	y3	326.1528	MPP
4	y4	435.2151	ILAH
5	y4	423.2004	MPPP
6	y5	560.2647	MHPPP
7	y6	673.3541	MHIPPP
8	y7	786.4356	MHILPPP
9	$[M+H]^+$	857.4730	MHILAPPP

Fig S4. Tandem mass spectrometry of novel cyclic peptides in *Amanita pallidorosea*. Manual analysis of amino acid compositions of Cyclo(EFIVFGIFP) (A), Cyclo(FVIIPPFIFP) (B), Cyclo(YFFNDHPP) (C), Cyclo(TIHLFSAP) (D), and Cyclo(MHILAPPP) (E), with inserted tables of matched amino acids; immonium ions were shown when present. The determined cyclic peptides were shown as circles.
