# Supplementary material for: Novel Cyclic Peptides from Lethal Amanita Mushrooms through a Genome-Guided Approach
Source: J Fungi (Basel). 2021 Mar 11;7(3):204. doi: 10.3390/jof7030204 (PMC7998459; doi:10.3390/jof7030204)
Supplement: Supplementary file 1 [file jof-07-00204-s001.zip › Additional file 3.pdf]

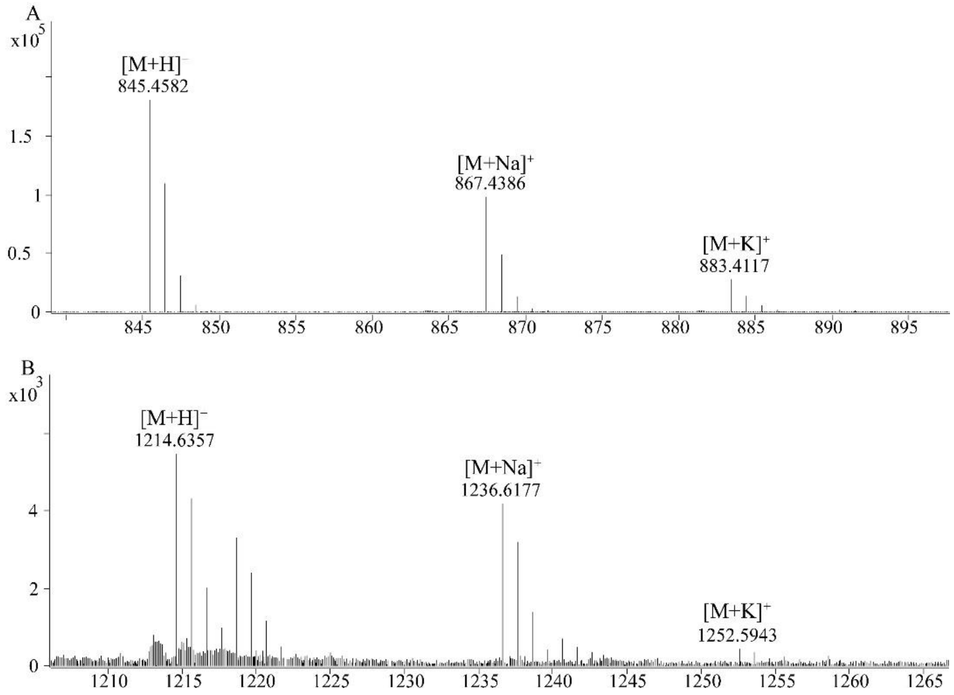

Fig S5. The monoisotopic masses of candidate new cyclic peptide in *Amanita exitialis.* The two molecules corresponded to the cyclized core peptide sequences of FVFVASPP (A) and LFFPPDFRPP (B). The adduct ions of $[M+Na]^+$ and $[M+K]^+$ were shown in each section.

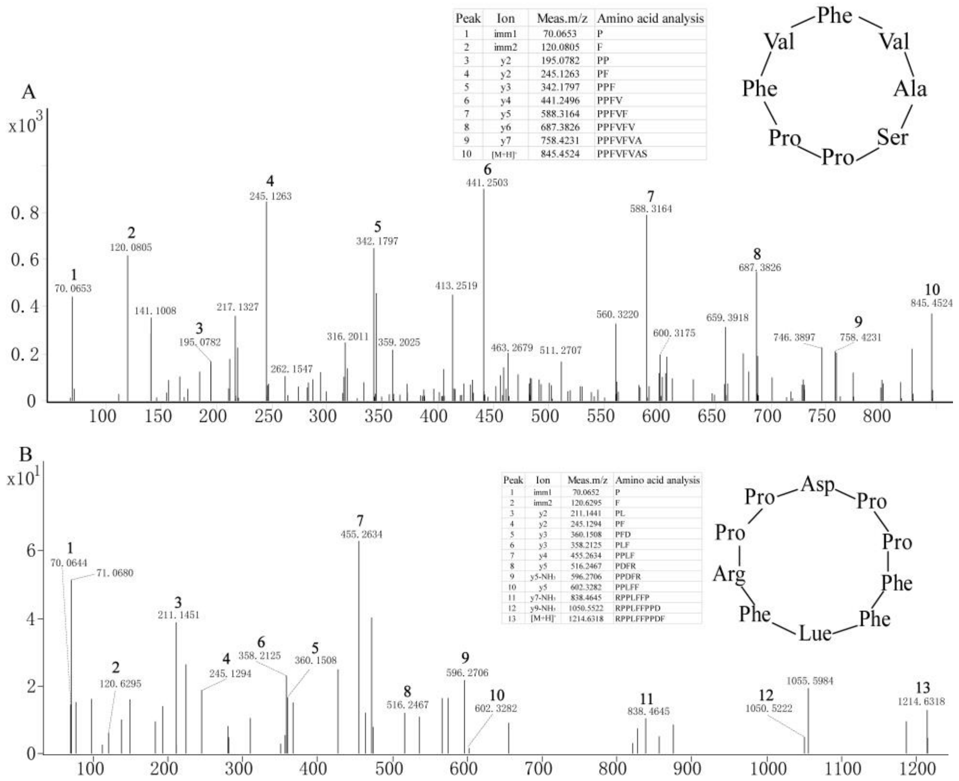

Peak	Ion	Meas.m/z	Amino acid analysis
1	imm1	70.0653	P
2	imm2	120.0805	F
3	y2	195.0782	PP
4	y2	245.1263	PF
5	y3	342.1797	PPF
6	y4	441.2496	PPFV
7	y5	588.3164	PPFVF
8	y6	687.3826	PPFVFV
9	y7	758.4231	PPFVFVA
10	$[M+H]^+$	845.4524	PPFVFVAS

Peak	Ion	Meas.m/z	Amino acid analysis
1	imm1	70.0652	P
2	imm2	120.6295	F
3	y2	211.1441	PL
4	y2	245.1294	PF
5	y3	360.1508	PFD
6	y3	358.2125	PLF
7	y4	455.2634	PPLF
8	y5	516.2467	PDFR
9	y5-NH_3	596.2706	PPDFR
10	y5	602.3282	PPLFF
11	y7-NH_3	838.4645	RPPLFFP
12	y9-NH_3	1050.5522	RPPLFFPPD
13	$[M+H]^+$	1214.6318	RPPLFFPPDF

Fig S6. Tandem mass spectrometry of novel cyclic peptides in *Amanita exitialis*. Manual analysis of

amino acid compositions of Cyclo(FVFVASPP) (A) and Cyclo(LFFPPDFRPP) (B), with inserted tables of matched amino acids; immonium ions were shown when present. The determined cyclic peptides were shown as circles.
