# Supplementary material for: Novel Cyclic Peptides from Lethal Amanita Mushrooms through a Genome-Guided Approach
Source: J Fungi (Basel). 2021 Mar 11;7(3):204. doi: 10.3390/jof7030204 (PMC7998459; doi:10.3390/jof7030204)
Supplement: Supplementary file 1 [file jof-07-00204-s001.zip › Additional file 4.pdf]

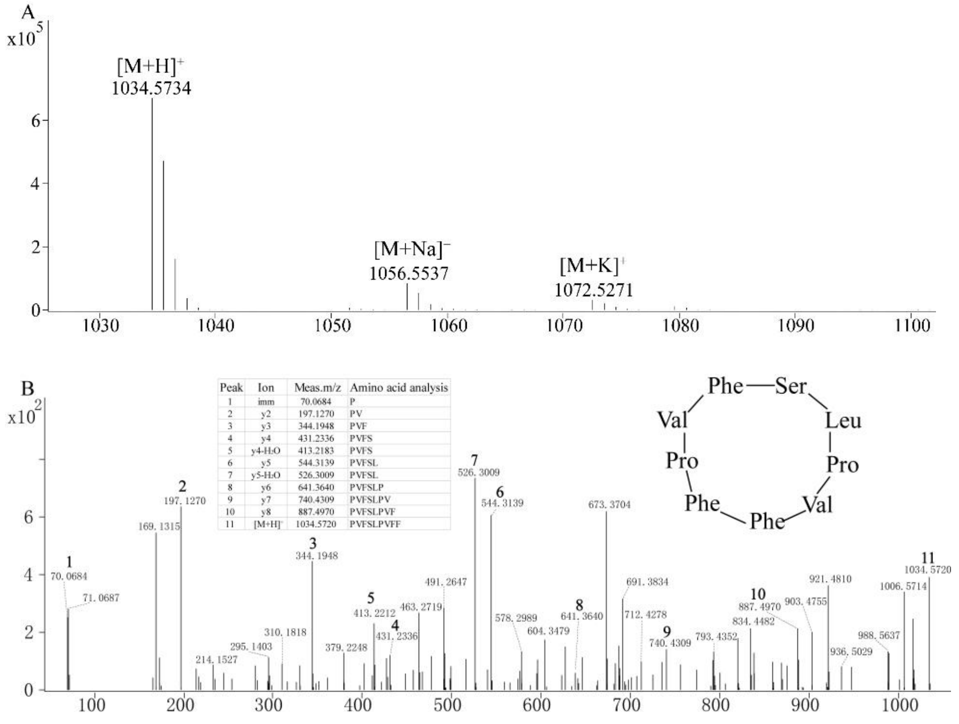

Peak	Ion	Meas.m/z	Amino acid analysis
1	imm	70.0684	P
2	y2	197.1270	PV
3	y3	344.1948	PVF
4	y4	431.2336	PVFS
5	y4-H_2O	413.2183	PVFS
6	y5	544.3139	PVFSL
7	y5-H_2O	526.3009	PVFSL
8	y6	641.3640	PVFSLP
9	y7	740.4309	PVFSLPV
10	y8	887.4970	PVFSLPVF
11	$[M+H]^+$	1034.5720	PVFSLPVFF

Fig S7. Mass spectrometry of amanexitide from *Amanita exitialis*. (A) The molecule corresponded to amanexitide was shown with adduct ions. (B) Manual analysis of amino acid composition of amanexitide with inserted tables of matched amino acids; immonium ions were shown when present. The determined cyclic peptide was shown as a circle.
