# Supplementary material for: Novel Cyclic Peptides from Lethal Amanita Mushrooms through a Genome-Guided Approach
Source: J Fungi (Basel). 2021 Mar 11;7(3):204. doi: 10.3390/jof7030204 (PMC7998459; doi:10.3390/jof7030204)
Supplement: Supplementary file 1 [file jof-07-00204-s001.zip › Additional file 5.pdf]

Table S1. MSDIN family and novel cyclic peptides in *Amanita pallidorosea*

Leader Peptide	Core Peptide	Recognition Sequence	Monoisotopic Mass
MSDINATRLP	**IWGIGCNP**	CVGDEVTALITRGEA	918.3541 (α-amanitin)
MSDINATRLP	**IWGIGCNP**	CVGDEVAALLTRGEA	918.3541 (α-amanitin)
MSDINATRLP	**IWGIGCDP**	CVGDDVTGVLTRGEA	919.3382 (β-amanitin)
MSDVNATRLP	**AWLVDCP**	CVGDDINRLLTRGEK	846.3217 (phallacidin)
MSDINATRLP	**AWLMTCP**	CVGDDVNPTLTRGE	802.3506
MSDINASRLP	**AWLATCP**	CAGDDVNPTLTRGE	788.3160 (phalloidin)
MSDINATRLP	**DPRRLLIP**	GGSDDVDSALTRDE	960.5869
MSDINTARLP	**EFIVFGIFP★**	CVGDDIQTVLTRGE	1049.5586
MSDINASRLP	**FFPEVGFFP**	CVGDDTNPILTRG	1067.5117
MSDINTIRVP	**FPWTGPFVP**	CVGDDVGSVLTHGE	1028.5120
MSDINTARLP	**FVIIPPFIFP★**	CVSDDIEMVLTRGE	1170.6841
MSDINATRLP	**HPFPLGLQP**	CAGDVDNLTLTRGE	986.5338
MSDINATRLP	**HPFPLGLQP**	CAGDVDNLTLTRGE	986.5338
MSDINAIRAPLP	**IFSLNP**	CVGDDVEVL	671.3643
MSDINVIRLP	**IFIYFP**	CVGDNIDNTLT	780.4210
MSDINAARLP	**LVYMILFP**	SVGDDIDVVLGRGE	976.5456
MSDINATRLP	**LGRPESLP**	CVGDDVNYILVSG	849.4709
MSDINTARLP	**MHILAPPP★**	CVSDDIEMVLTRGE	856.4629
MSDINATRLP	**NWHAGPTRPP**	CVADDVSLTLTRGE	1113.5468
MSDINAARLP	**NLFVWIPP**	CISDDINSTLTRGE	966.5327
MSDINTARLP	**VFFMPPFIPP**	CVSDDIQMVLTRGEK	1172.6093
MSDINTTRLP	**YFFNDHPP★**	CASDDIQMVFTRGE	1017.4345
MSDVNATRLP	**MAFPEFLA**	CVGDDVNHTLTRGE	906.4310
MSDNASRLP	**YFFGFTP**	SVGDDVNPTLTRGE	859.3905
MSDTNATRLP	**SIFIVYPP**	CVSDDVNSTLTRGE	916.5058
MSDMNVVRLP	**ISDPTAYP**	CVGDDIQAVLGRGE	844.3967
MSDMNVVRLP	**ISDPTAYP**	CVGDDIQAVLGRGE	844.3967
MSDMNTACLP	**IFIVFPIPP**	CVSDDIQTVLTRGE	1023.6157
MSDLNATRLP	**FNLFRFPYP**	CIGDDSGSVLTLGEG	1181.6022
MSETNAARLP	**TIHLFSAP★**	SVGDDIEVVLGRGE	866.4650
MSNINTARLP	**FLVPSFPP**	CVSDDIQIVLTRGEK	884.4796
MADINASRLP	**LNILPFHLPP**	CVSDDATSTLTRGE	1141.6648
MYDINTTRLP	**HFFNLTPP**	CVRDDI	953.4759

[1]★and underlined letters indicate novel cyclic peptides detected with MS and MS/MS.

Predicted monoisotopic masses of cyclic peptides with no further modifications.

[2]The monoisotopic masses are for unmodified cyclic peptides based on MSDIN core peptides, except for major toxins.
